# Conformational Analysis of Geometric Isomers of Pitavastatin Together with Their Lactonized Analogues

**DOI:** 10.3390/molecules181113283

**Published:** 2013-10-28

**Authors:** Damjan Makuc, Jan Fabris, Zdenko Časar, Janez Plavec

**Affiliations:** 1 Slovenian NMR Centre, National Institute of Chemistry, Hajdrihova 19, Ljubljana SI-1000, Slovenia; E-Mail: damjan.makuc@ki.si; 2 EN-FIST Centre of Excellence, Dunajska cesta 156, Ljubljana SI-1000, Slovenia; 3 Cadonic Consultancy Services, LL.C., Cesta na postajo 74, Brezovica pri Ljubljani SI-1351, Slovenia; E-Mail: jan.fabris@sandoz.com; 4 Sandoz Development Center Slovenia, API Development, Organic Synthesis Department, Lek Pharmaceuticals, d.d., Kolodvorska 27, Mengeš SI-1234, Slovenia; E-Mail: zdenko.casar@sandoz.com; 5 Sandoz GmbH, Global Portfolio Management API, Biochemiestrasse 10, Kundl A-6250, Austria; 6 Faculty of Pharmacy, University of Ljubljana, Aškerčeva cesta 7, Ljubljana SI-1000, Slovenia; 7 Faculty of Chemistry and Chemical Technology, University of Ljubljana, Aškerčeva cesta 5, Ljubljana SI-1000, Slovenia

**Keywords:** conformational analysis, intramolecular dynamics, NMR studies, barriers to rotation, super-statins

## Abstract

Super-statins are synthetic inhibitors of 3-hydroxy-3-methylglutaryl-coenzyme A reductase, which is the rate-limiting enzyme responsible for the biosynthesis of cholesterol. All of the super-statins with a C=C double bond spacer between the heterocyclic and the dihydroxycarboxylic moiety that are currently on the market exist as *E*-isomers. To extend the understanding of conformational and thermodynamic preferences of *Z*-isomeric super-statin analogues, this study focused on analyzing pitavastatin and its lactonized derivatives via NMR spectroscopy and *ab initio* calculations. *Z*-isomeric pitavastatin analogues exist in solution as a pair of interconverting rotamers, where the Gibbs free energies between the major and minor rotamers are within 0.12 and 0.25 kcal mol^−1^ and the rotational energy barriers are between 15.0 and 15.9 kcal mol^−1^. The analysis of long-range coupling constants and *ab initio* calculations revealed that rotation across the C5'–C7 single bond is essential for generating a pair of atropisomers. The overall comparison of the results between *Z*-isomeric pitavastatin and rosuvastatin analogues demonstrated that the former are to some extent more flexible to attain numerous conformations. Demonstrating how structural differences between super-statin analogues induce distinctive conformational preferences provides important insight into the super-statins’ conformational variability and may well improve future drug design.

## 1. Introduction

Super-statins are fully synthetic derivatives of 3-hydroxy-3-methylglutaryl-coenzyme A reductase (HMGR; EC 1.1.1.88) inhibitors [1]. HMGR is the rate-limiting enzyme responsible for the biosynthesis of cholesterol [2]. They consist of a chiral 3,5-dihydroxyhept-6-enoic or -heptanoic acid side-chain attached to a heterocyclic core. Four of the super-statins are currently on the market: fluvastatin [3], atorvastatin [4], rosuvastatin [5], and pitavastatin [6]. Except for atorvastatin, all of them contain the C=C double bond as a spacer between the heterocyclic core and the dihydroxycarboxylic moiety and all of them exist as *E*-oriented geometric isomers. In the early discovery stage of super-statins it was shown that some of their *Z*-isomeric analogues showed only weak [7] or even none [8] *in vitro* inhibitory activity on HMGR, however, no further studies were conducted to explain these observations. X-ray crystal structures of human HMGR complexed with several *E*-isomeric statins as well as kinetic and thermodynamic parameters of their binding and inhibition of HMGR enabled detailed characterization of structural mechanism of inhibition [9–11]. Nevertheless, some biologically active compounds and drugs with *Z*-configuration across the exocyclic C=C bond exhibit better pharmacodynamic properties or different activity than their *E*-isomeric counterparts. Such compounds are for example combretastatins [12,13], prostaglandins [14], and recently norendoxifen [15].

**Figure 1:**
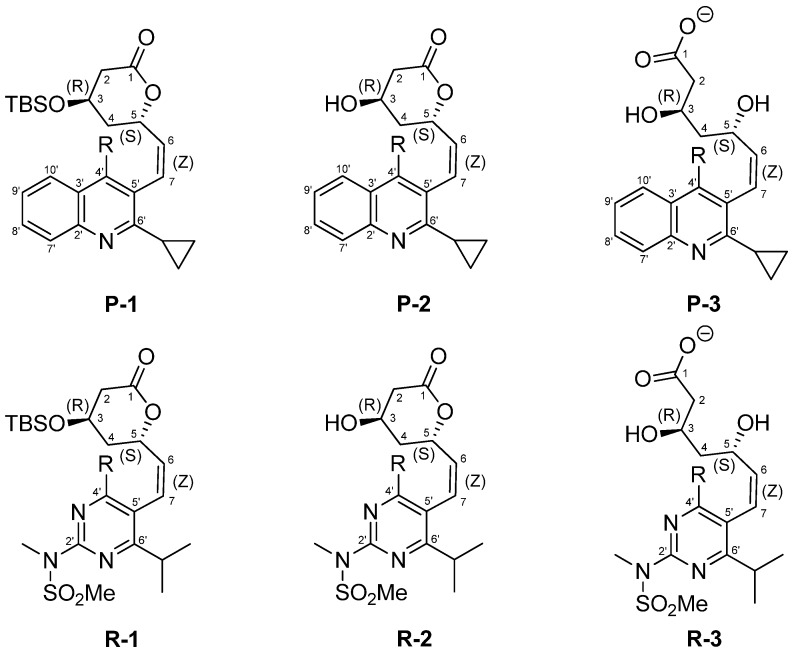
Chemical structures of *Z*-isomeric pitavastatin (**P-1**, **P-2**, **P-3**) and rosuvastatin analogues (**R-1**, **R-2**, **R-3**), together with atom numbering for 4-*O*-TBS protected lactone **1**, deprotected lactone **2**, and calcium salt **3** of the corresponding super-statin analogue (R = 4-F–C_6_H_4_).

In our latest research we studied the conformational behavior of *E*/*Z*-isomeric pairs of rosuvastatin and its lactonized analogues, where we showed that ^1^H-NMR resonance line broadening observed at room temperature originates from the dynamic exchange between the two rotamers of the *Z*-isomeric rosuvastatin compounds **R-1**, **R-2**, and **R-3** (Figure 1) [16]. The two rotamers showed well-defined differences in ^4^*J*_H5-H7_ allylic coupling constants and in NOESY cross-peaks between major and minor conformers, which suggested the presence of two rotamers along the C5–C6 single bond. Furthermore, two conformers observed in the NMR spectra at lowered temperature corresponded to a pair of atropisomers, where concerted rotation along both C5–C6 and C5'–C7 bonds was supported by experimentally determined as well as by calculated rotational energy barriers. On the other hand, the corresponding *E*-isomeric rosuvastatin and its analogues showed only a single set of narrow resonances in NMR spectra, which demonstrated that they exist only as a single conformer.

Fluvastatin exists on the market as a racemic mixture and therefore could not be directly comparable with other super-statins, which are enantiomerically pure. Atorvastatin contains the C-C single bond as a spacer between the heterocyclic and the dihydroxycarboxylic moiety and so could not form the *E*/*Z*-geometric isomers. Therefore, the only one left of the marketed super-statins, which may show intriguing conformational properties, is pitavastatin. In one of our recent work we studied the synthetic pathway towards pitavastatin using lactonized statin side-chain precursor [17]. During that research the *Z*-isomeric 4-*O*-TBS protected pitavastatin lactone **P-1** (Figure 1) was isolated. Compound **P-1** represented the major side product in the Wittig reaction between phosphonium salt of an appropriately functionalized heterocyclic moiety and lactonized statin side chain precursor [18–20]. The characterization of **P-1** with NMR spectroscopy showed similar line broadening in ^1^H NMR spectra as was noticed in our rosuvastatin study. Therefore, both the deprotected *Z*-isomeric pitavastatin lactone **P-2** and the *Z*-isomeric analogue of pitavastatin calcium **P-3** (Figure 1) were readily synthetized.

The conformational behavior of all three *Z*-isomeric pitavastatin analogues **P-1**, **P-2**, and **P-3** were explored and compared to *Z*-isomeric rosuvastatin analogues **R-1**, **R-2**, and **R-3**. They both possess the same chiral dihydroxycarboxylic moiety and 4-fluorophenyl group attached to the heterocyclic moiety at C5' and C4', respectively. The heterocyclic moiety and the corresponding propyl group attached to it represent the main structural differences between pitavastatin and rosuvastatin analogues. Pitavastatin derivatives contain quinoline as a heterocyclic core and the cyclopropyl group attached to C6', whereas rosuvastatin derivatives contain pyrimidine with the isopropyl group attached to C6' and the *N*-methylmethanesulfonamide group to C2' (Figure 1). We hypothesized that these structural characteristics could have significant impact on their individual conformational properties, which were elucidated using NMR spectroscopy and *ab initio* calculations. Investigating conformational preferences of *Z*-isomeric statin analogues may also explain why their *E*-isomeric counterparts do not exhibit broadened resonances in NMR spectra.

## 2. Results and Discussion

**Scheme 1:**
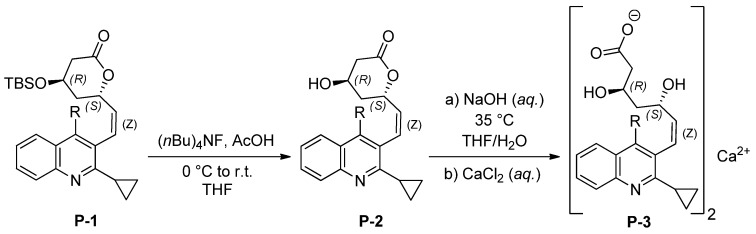
Synthesis of *Z*-isomers of pitavastatin analogues.

Compound **P-1** was synthesized and isolated according to the procedure in our previous work [17]. Additionally, **P-2** was prepared via the deprotection of **P-1** with tetrabutylammonium fluoride trihydrate and acetic acid at 0 °C. **P-2** was then treated with aqueous NaOH and CaCl_2_ to give *Z*-isomeric pitavastatin as a calcium salt **P-3** (Scheme 1).

The assignment of signals in ^1^H- and ^13^C-NMR spectra for all three pitavastatin analogues was achieved from signal multiplicities, integral values and chemical shifts as well as from the correlations in 2D DQF-COSY, ^1^H–^13^C heteronuclear HSQC and HMBC spectra. Synthetic procedures and full spectroscopic characterizations of newly synthesized **P-2** and **P-3** are reported in the Experimental Section.

**Figure 2:**
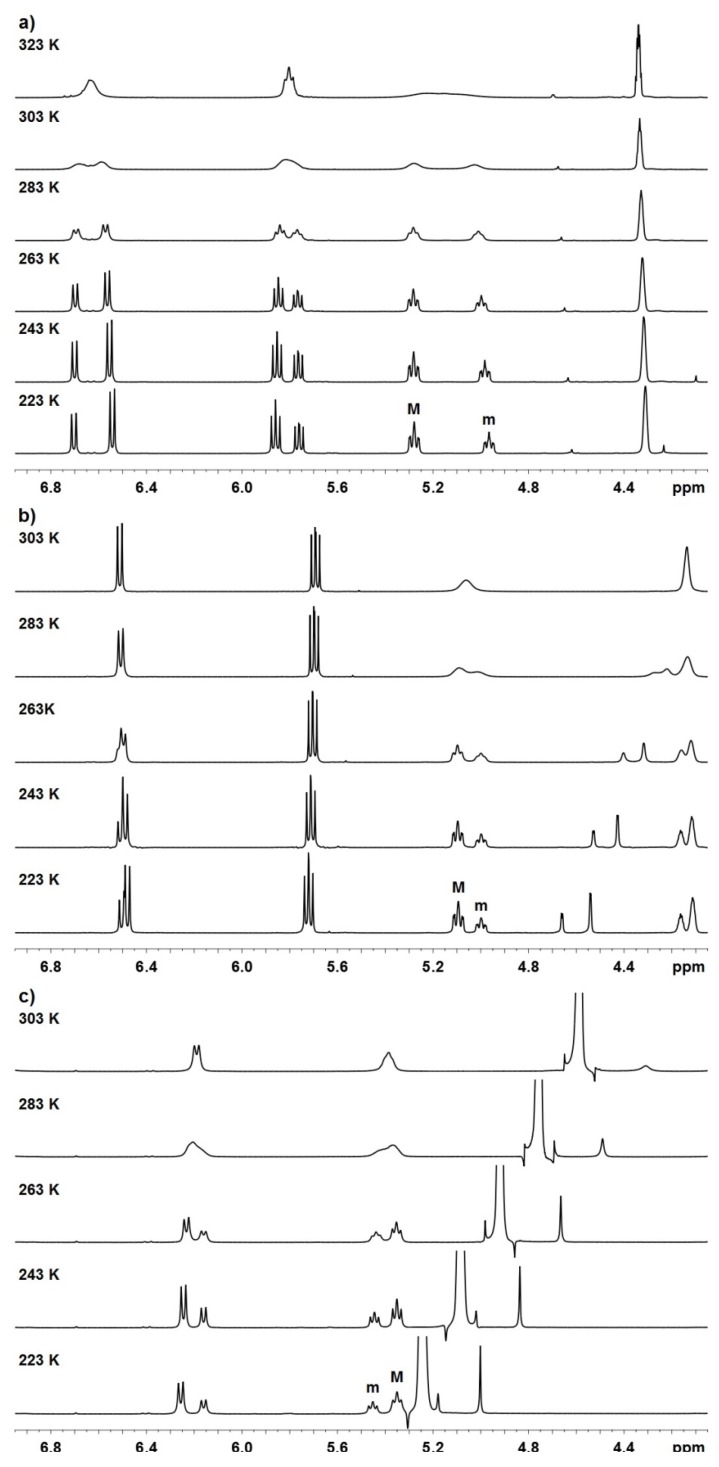
^1^H-NMR (600 MHz) spectra of (**a**) **P-1** in acetone-*d*_6_ in the range from 223 to 323 K; (**b**) **P-2** in acetone-*d*_6_ in the range from 223 to 303 K; (**c**) **P-3** in methanol-*d*_4_ in the range from 223 to 303 K.

All three *Z*-isomeric pitavastatin derivatives showed a single set of very broad NMR resonances (e.g., Δ*ν_1/2_* ≈ 9 Hz for H7 of **P-3** at 303 K), whereas the corresponding *E*-isomeric pitavastatin analogues showed a single set of narrow NMR resonances (e.g. Δ*ν_1/2_* ≈ 2 Hz for H7 of *E*-isomeric pitavastatin calcium at 303 K). *E*-isomeric pitavastatin analogues show a single set of resonances even upon cooling to 223 K, which means that they do not exhibit several conformations. In order to acquire NMR spectra at low temperature and to enable the comparison of spectra between pitavastatin and rosuvastatin derivatives, **P-1** and **P-2** were dissolved in acetone-*d*_6_ and **P-3** in methanol-*d*_4_. Step by step cooling of the samples resulted in two sets of sharp and well-resolved signals in ^1^H-NMR spectra. The ratios between the major (M) and minor (m) set of signals for **P-1**, **P-2**, and **P-3** are between ca. 1.4:1 and 2.0:1 at 223 K. Arrays of temperature-dependent ^1^H NMR spectra of **P-1**, **P-2**, and **P-3** are shown in Figure 2.

Well-resolved proton signals at 223 K enabled determination of ^3^*J*_H5-H6_, ^3^*J*_H6-H7_ and ^4^*J*_H5-H7_ coupling constants. ^3^*J*_H5-H6_ coupling constants for the major and minor conformers of **P-1** are 10.2 Hz and 9.1 Hz, respectively. Unfortunately, we were unable to determine the corresponding values for **P-2** and **P-3** due to broad multiplet observed in the ^1^H-NMR spectra. ^3^*J*_H6-H7_ coupling constants for the major and minor conformers of **P-1**, **P-2**, and **P-3** are between 11.2 and 11.3 Hz along with 11.3 and 11.4 Hz, respectively. The values of all of the three-bond coupling constants for the major and minor conformers of pitavastatin analogues are hence very comparable to the corresponding ones of rosuvastatin analogues. This could as well propose two probable conformations with torsion angles *θ* [H5–C5–C6–H6] of 0° ± 30° and 180° ± 30° [21]. Interestingly, all ^4^*J*_H5-H7_ coupling constants for pitavastatin analogues are 0.0 Hz, which implied that the preferred orientation along the C5–C6 bond is *anti* (*θ* ~ 180°). On the contrary, ^4^*J*_H5-H7_ coupling constants of 1.1 and 0.8 Hz were observed for the minor conformers of rosuvastatin analogues **R-1** and **R-2**, respectively, which suggested that the rotation along the C5–C6 bond allows generating a pair of rotamers in rosuvastatin analogues [16]. These observations could be explained by the structural differences that exist between rosuvastatin and pitavastatin analogues, where the latter contain the cyclopropyl instead of the isopropyl group, which is sterically smaller and consequently less likely to hinder the rotation.

**Figure 3:**
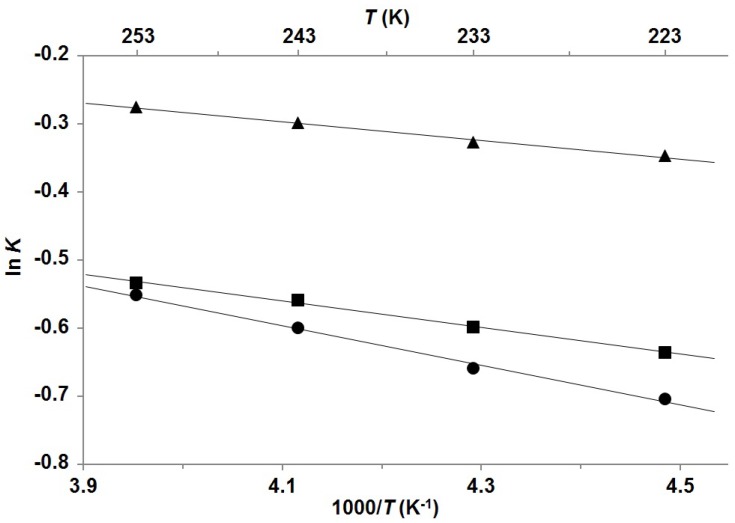
Van’t Hoff plots for dynamic conformational equilibrium M ⇄ m of **P-1** (▲), **P-2 **(■), and **P-3 **(●) in the temperature range from 223 K to 253 K. The straight lines are the best fits to the experimental data using least-square method. Pearson correlation coefficient R^2^ was 0.9918, 0.9965, and 0.9941 for **P-1**, **P-2**, and **P-3**, respectively.

Gradual cooling of **P-1**, **P-2**, and **P-3** from 253 to 223 K with 10 K steps allowed thermodynamic examination of the conformational equilibrium M ⇄ m. The average values of integrals of at least two different well-resolved proton signals were considered for the estimation of mole fractions of the minor conformers and equilibrium constants (for details see Table S1 in Supplementary Materials). Through the determination of the temperature-dependent ratios between both conformers van’t Hoff plots were created (Figure 3) and hence thermodynamic parameters for **P-1**, **P-2**, and **P-3** were calculated (Table 1). In all cases, the increase of temperature resulted in increased population of the minor conformer with respect to the major conformer. Mole fractions of the minor conformers in that temperature range lie between 0.33 and 0.43 and are significantly greater for **P-1**, **P-2**, and **P-3** than the corresponding ones for **R-1**, **R-2**, and **R-3**, which are between 0.12 and 0.31 [16]. Consequently, the enthalpy contributions for pitavastatin analogues are smaller (between 0.27 and 0.58 kcal mol^−1^) and therefore more comparable with their entropy contributions (between 0.14 and 0.35 kcal mol^−1^) at room temperature. Values of Gibbs free energies are hence more close to zero (between 0.12 and 0.25 kcal mol^−1^), which propose that the energy differences between both interconverting conformers are smaller for pitavastatin derivatives. On the other hand, it is noteworthy that the Gibbs free energies for rosuvastatin analogues are significantly greater, between 0.35 and 0.53 kcal mol^−1^ [16]. Remarkably, all obtained results are similar for pitavastatin derivatives but differ considerably when compared with results for rosuvastatin derivatives. It could be expected that if only rotation along C5'–C7 bond contributes to the formation of two conformers, the difference of Gibbs free energy between the formed atropisomers would be quite small. These results implicate that in the cases of pitavastatin analogues there is only one rotatable bond (C5'–C7) crucial for the formation of two rotamers, whereas in the cases of rosuvastatin analogues there are two such rotatable bonds (C5–C6 and C5'–C7).

**Table 1:** Conformer population and thermodynamic parameters for equilibrium M ⇄ m.

Compound	Mole fractions of minor conformers			Thermodynamic parameters ^a^			
	223 K	233 K	243 K	253 K	Δ *H*°	– *T*Δ*S*°	Δ *G*°
**P-1** ^b^	0.414	0.419	0.426	0.432	0.27	−0.16	0.12
**P-2** ^b^	0.346	0.355	0.364	0.370	0.39	−0.14	0.25
**P-3** ^c^	0.331	0.341	0.355	0.366	0.58	−0.35	0.23

^a^ Reported in kcal mol^−1^; ^b^ Compound dissolved in acetone-*d*_6_; ^c^ Compound dissolved in methanol-*d*_4_.

To support our claims, 2D NMR NOESY spectra with mixing times of 80 and 200 ms were recorded at 253 K. For all three compounds **P-1**, **P-2**, and **P-3** merely the NOESY cross-peaks of the same proton signals between major and minor conformers were observed (e.g., H-7 signal of major conformer exhibits NOESY cross-peak with H-7 signal of minor conformer), which suggested that dynamic exchange between rotamers prevented the detailed study of their conformational properties by NOESY experiments.

The estimation of chemical shift differences Δ*ν* of separated signals and coalescence temperature for **P-1**, **P-2**, and **P-3** enabled the determination of the rate constant for interconversion *k*_E_ and the rotational energy barrier Δ*G*^‡^ using the Eyring equation. The results showed that rotational energy barriers lie between 15.0 and 15.9 kcal mol^−1^ (Table 2) and are very comparable to the corresponding values for **R-1**, **R-2**, and **R-3**, which are between 14.4 and 14.8 kcal mol^−1^ [16]. The order of magnitude of rotational energy barriers suggested that in the case of pitavastatin analogues the rotation across the C5'–C7 bond, which generates a pair of atropisomers, is significant for producing two sets of signals, which were observed in NMR spectra at lowered temperature.

**Table 2:** Interconversion rate constants *k*_E_ and rotational energy barriers Δ*G*^‡^.

Compound	*T*_coal._ (K)	Δ*ν* (Hz)	*k*_E_ (s^−1^)	Δ*G*^‡^ (kcal mol^-1^)
**P-1** ^a^	323	96.4	214	15.6
**P-2** ^a^	303	14.4	32	15.9
**P-3** ^b^	303	57.3	127	15.0

^a^ Compound dissolved in acetone-*d*_6_; ^b^ Compound dissolved in methanol-*d*_4_.

**Figure 4:**
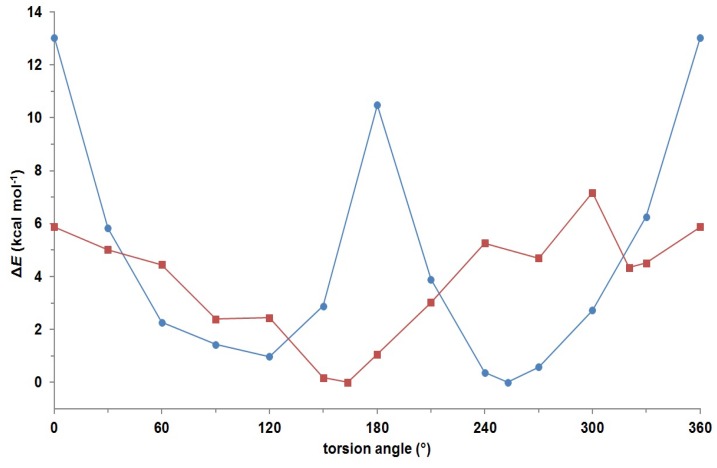
Relative potential energy profile of **P-1** as a function of the torsion angles ϕ [C6'–C5'–C7–H7] (●) and *θ* [H5–C5–C6–H6] (■) at the B3LYP/6-311+G(d,p) level of theory.

**Figure 5:**
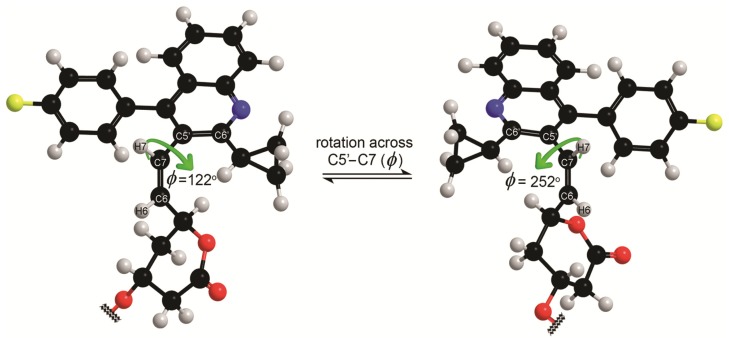
The energetically minimized preferred conformations of **P-1** at the B3LYP/6-311+G(d,p) level of theory. For clarity, the *tert*-butyldimethylsilyl group attached to C3 is not presented (although it was included in calculations).

The experimentally determined rotational energy barrier for **P-1** was supported by quantum mechanical calculations at B3LYP/6-311+G(d,p) level of theory using Gaussian 09 program [22]. The torsion angle ϕ [C6'–C5'–C7–H7] was defined to follow energetic variations induced by reorientation around the C5'–C7 bond with 30° resolution. The relative potential energy profile of **P*-*1** as a function of the torsion angle ϕ is shown in Figure 4. The two energetically optimized minima at ϕ = 122° and ϕ = 252° suggested presence of a pair of atropisomers with the energetic difference of 0.98 kcal mol^−1^ between them. Energetically minimized preferred conformations, that were calculated for **P-1** with respect to rotation across the C5'–C7 single bond, are shown in Figure 5. Rotational energy barrier along C5'–C7 bond is 13.0 kcal mol^−1^, which is in good agreement with the experimentally determined rotational energy barrier (Table 2).

Additionally, the rotation across the C5–C6 bond was also examined by *ab initio* calculations at the same level of theory, where the torsion angle *θ* [H5–C5–C6–H6] was defined. The results revealed several energetic minima, where the global minimum is found at *θ* = 164° (Figure 4). This observation confirmed *anti* orientation along the C5–C6 bond, which supports our findings based on long-range coupling constants. The rotational energy barrier of 7.2 kcal mol^−1^ suggests that the rotation across the C5–C6 bond is less sterically hindered than across the C5'–C7 bond.

The study of *Z*-isomeric pitavastatin analogues revealed some interesting insights related to conformational preferences in **P-1**, **P-2**, and **P-3** compared to rosuvastatin analogues. Even though both of the *Z*-isomeric statin analogues have many common structural properties and both exist as a pair of interconverting atropisomers, there are still some variances that induce certain differences in their conformational behavior. The cyclopropyl group of pitavastatin analogues is sterically smaller than the isopropyl group of rosuvastatin analogues, which also have the *N*-methylmethanesulfonamide group attached to the heterocyclic core. Rosuvastatin analogues are therefore exposed to additional steric crowding, for which the rotation across several bonds is more hindered. Most notably, steric hindrance prevents the full rotation across the C5'–C7 bond in **R-1** [16]. On the other hand, pitavastatin derivatives are obviously structurally less complex and so to some extent more flexible to attain various conformations. Interestingly, *E*-isomeric pitavastatin and rosuvastatin analogues do not exhibit dynamic equilibrium between rotamers, which suggests that the orientation across the C=C double bond represents crucial factor for generating a pair of conformers. Moreover, the results presented herein in conjunction with our previous study [16] show what effect can a structural change have on conformational preferences, which provide important insight in the super-statins’ conformational variability and may well improve future drug design.

## 3. Experimental

### 3.1. General

Reagents and solvents were acquired from commercial sources and used without further purification. Reactions were monitored using analytical TLC plates (Merck, Darmstadt, Germany; silica gel 60 F254, 0.25 mm), and compounds were visualized with UV radiation. Silica gel grade 60 (70–230 mesh, Merck) was used for column chromatography. Melting points were determined with a Mettler Toledo DSC822^e^ apparatus (Mettler-Toledo GmbH, Schwarzenbach, Switzerland), with the heating rate of 10 °C min^−1^ and are referred to as onset values and peak values. Optical rotations were measured on a Perkin Elmer 341-series polarimeter (Perkin Elmer, Waltham, MA, USA). IR spectra were recorded on a Thermo Nicolet Nexus FTIR spectrometer (Thermo, Madison, WI, USA) and only noteworthy absorptions are listed. High-resolution mass spectra were obtained with a VG-Analytical AutospecQ instrument (VG Analytical, Manchester, UK) and a Q-TOF Premier instrument (Waters Corporation, Milford, MA, USA).

### 3.2. NMR Experiments

^1^H- and ^13^C-NMR spectra were acquired on Agilent Technologies (Agilent Technologies Santa Clara, CA, USA) VNMRS 600 MHz, Unity Inova 300 MHz and DD2 300 MHz NMR spectrometers. Sample concentrations used in NMR studies were ca. 20 mM dissolved in acetone-*d*_6_ or methanol-*d*_4_. Two-dimensional homonuclear (COSY) and heteronuclear (HSQC, HMBC) NMR experiments with gradients were used to structurally elucidate pitavastatin analogues. 2D NOESY experiments were performed using a mixing times of 80 and 200 ms, which ensures the operation in the initial linear part of the NOESY buildup curve.

### 3.3. Ab Initio Calculations

Initial structures were generated by Chem3D Pro 10.0 software and energy minimization at B3LYP/6-311+G(d,p) level was performed using Gaussian 09 [22]. Torsion angles *θ* [H5–C5–C6–H6] and ϕ [C6'–C5'–C7–H7] were defined to follow energetic changes induced by reorientation. The relative energy profile of the torsion angles *θ* and ϕ were calculated with 30° resolution, where orientations were restrained along [H5–C5–C6–H6] and [C6'–C5'–C7–H7] torsion angles, respectively, while other degrees of freedom were freely optimized. In addition, calculations were carried out without any constraints for each (local) minimum-energy conformation. Freely optimized conformations exhibited local minimum at torsion angle ϕ = 122° (0.9 kcal mol^–1^) and global minimum at ϕ = 252° (0.0 kcal mol^–1^). Conformations exhibiting (local) minima at torsion angle *θ* = 90°, 150° and 270° all minimized to 164° (0.0 kcal mol^–1^); an additional local minimum was found at *θ* = 321° (4.3 kcal mol^–1^). Frequency calculations verified that the optimized geometries at (local) minima were stable points on the potential energy surface.

### 3.4. Synthetic Procedures

*(4R,6S)-6-((Z)-2-(2-Cyclopropyl-4-(4-fluorophenyl)quinolin-3-yl)vinyl)-4-hydroxytetrahydro-2H-pyran-2-one* (**P-2**). A solution of Bu_4_NF∙3H_2_O (420 mg, 1.33 mmol; 2.3 equiv) and AcOH (219 µL, 3.83 mmol; 6.6 equiv) in THF (2.5 mL) was cooled in ice bath. Then a solution of **P-1** (300 mg, 0.58 mmol) in THF (2.5 mL) was added. The solution was left to stir for 24 h at room temperature. The solvent was evaporated under reduced pressure and the residue was dissolved in EtOAc (5 mL). The organic layer was washed with water (5 mL), saturated solution of NaHCO_3_ (5 mL), brine (5 mL) and water (5 mL) respectively. Organic layer was dried over Na_2_SO_4_ and evaporated under reduced pressure. The residue was purified by column chromatography (silica gel, EtOAc–hexane, 1:1) to give 170 mg (73% yield) of **P-2**. ^1^H-NMR (600 MHz, acetone-*d*_6_): 7.91 (1H, m, Ar), 7.67 (1H, m, Ar), 7.54–7.24 (6H, m, Ar, 4-F–C_6_H_4_), 6.61 (1H, dd, *J = * 11.3, 0.7 Hz, H-7), 5.80 (1H, dd, *J = * 11.3, 9.5 Hz, H-6), 5.16 (1H, br s, H-5), 4.24 (2H, br s, H-3, OH), 2.61 (1H, dd, *J = * 17.4, 4.6 Hz, H-2a), 2.50 (1H, m, C*H*(CH_2_)_2_), 2.41 (1H, dd, *J* Hz, H-2b), 1.98–1.48 (2H, m, H-4a, H-4b), 1.29 (2H, br s, CH(C*H*_2_)_2_), 1.06 (2H, br s, CH(C*H*_2_)_2_). ^13^C-NMR (75 MHz, acetone-*d*_6_): 169.8 (C-1), 163.3 (d, *J = * 245.1 Hz, 4-F–C_6_H_4_), 161.4 (Ar), 148.2 (Ar), 145.8 (Ar), 134.0 (d, *J = * 3.3 Hz, 4-F–C_6_H_4_), 133.4 (C-6), 132.7 (br s, 4-F–C_6_H_4_), 130.1 (Ar), 129.9 (Ar), 129.7 (C-7), 128.7 (Ar), 126.53 (Ar), 126.49 (Ar), 126.4 (Ar), 116.3 (d, *J =* 21.6 Hz, 4-F–C_6_H_4_), 116.1 (d, *J =* 21.6 Hz, 4-F-C_6_H_4_), 73.4 (C-5), 63.0 (C-3), 39.2 (C-2), 35.9 (C-4), 15.9 (*C*H(CH_2_)_2_), 11.7 (br s, CH(*C*H_2_)_2_). IR (KBr): 3424, 3063, 3007, 2954, 2922, 1708, 1513, 1488, 1255, 1222, 1159, 1034, 764, 668, cm^−1^. Mp: 190.8 °C (onset), 192.7 °C (peak). HRMS (ESI): MH^+^, found: 404.1656. C_25_H_23_FNO_3_ requires 404.1656. ${[}_{\alpha }^{]}D25$: + 126.8° [*c* 0.90, CH_2_Cl_2_]. 

*(3R,5S,Z)-7-(2-Cyclopropyl-4-(4-fluorophenyl)quinolin-3-yl)-3,5-dihydroxyhept-6-enoate calcium* (**P-3**). To a solution of **P-2** (404 mg, 1.00 mmol) in THF–water, 4:1 (5 mL) at 35 °C was added 8M aq NaOH (138 µL, 1.075 mmol, 1.075 equiv). After 16 h the reaction was finished and THF was evaporated under reduced pressure. Sodium salt of *Z*-isomeric pitavastatin spontaneously precipitated from the aqueous solution. The suspension was cooled in an ice bath and THF (1 mL approx) was added drop by drop till all of the precipitate has dissolved again. To a concentrated solution of sodium salt of *Z*-isomeric pitavastatin was added a solution of CaCl_2_ (134 mg, 1.20 mmol; 1.2 equiv) in water (0.5 mL). Immediately occurred white precipitate was left to stir for 1 h. Then it was filtered off, washed with water (5 × 2 mL) and dried at 90 °C under vacuum to give 340 mg (77% yield) of **P-3**. ^1^H-NMR (600 MHz, methanol-*d*_4_): 7.90 (1H, m, Ar), 7.60 (1H, m, Ar), 7.49–7.12 (6H, m Ar, 4-F–C_6_H_4_), 6.42 (1H, d, *J = * 11.5 Hz, H-7), 5.62 (1H, m, H-6), 4.17 (1H, m, H-5), 3.94 (1H, m, H-3), 2.52 (1H, m, C*H*(CH_2_)_2_), 2.28–1.94 (2H, m, H-4a, H-4b), 1.80–0.72 (8H, m, H-2a, H-2b, CH(C*H*_2_)_2_, 2∙OH). ^13^C-NMR (75 MHz, methanol-*d*_4_): 181.8 (C-1), 163.9 (d, *J = * 245.9 Hz, 4-F–C_6_H_4_), 162.3 (Ar), 148.5 (Ar), 146.4 (br s, Ar), 137.3 (br s, C-6), 134.4 (d, *J = * 2.9 Hz, 4-F–C_6_H_4_), 133.1 (br s, 4-F–C_6_H_4_), 130.1 (Ar), 129.8 (Ar), 129.4 (Ar), 127.7 (C-7), 127.0 (Ar), 126.8 (Ar), 126.6 (Ar), 116.4 (d, *J = * 22.2 Hz), 116.3 (d, *J = * 22.1 Hz, 4-F–C_6_H_4_), 69.0 (C-3), 68.7 (C-5), 44.9 (C-4), 43.8 (C-2), 16.3 (*C*H(CH_2_)_2_), 11.6 (m, CH(*C*H_2_)_2_). IR (KBr): 3397, 3067, 3008, 2919, 1561, 1513, 1489, 1417, 1222, 1158, 844, 765, 560 cm^−1^. Mp:/(amorphous). HRMS (ESI): MH^+^, found: 422.1764. C_25_H_25_FNO_4_ requires 422.1762. ${[}_{\alpha }^{]}D25$: + 19.7° [*c* 0.98, MeOH] for sodium salt of pitavastatin **P-3**. ${[}_{\alpha }^{]}D25$: + 41.2° [*c* 0.25, DMSO] for calcium salt of pitavastatin **P-3**.

## 4. Conclusions

The *Z*-isomeric pitavastatin analogues **P-1**, **P-2**, and **P-3** were synthesized and characterized using various NMR spectroscopy techniques. Acquiring NMR spectra at lower temperature revealed that the resonance line broadening observed at room temperature originates from the dynamic exchange between two rotamers. Firstly, according to the ^4^*J*_H5-H7_ long-range coupling constants, the preferred orientation along the C5–C6 bond for pitavastatin analogues is *anti*. This orientation is in addition supported by *ab initio* calculations. Secondly, the determination of mole fractions of minor conformers for pitavastatin analogues, which were between 1.4 and 2.7 fold greater than for rosuvastatin analogues, enabled calculations of thermodynamic preferences. The values of Gibbs free energies for the equilibrium between the major and minor conformers of pitavastatin analogues at room temperature are consequently smaller than for rosuvastatin analogues. Finally, the experimentally established values of rotational energy barriers Δ*G*^‡^ for pitavastatin and rosuvastatin analogues are very comparable with each other and in good agreement with their calculated values. The presented results confirm that for *Z*-isomeric pitavastatin analogues the rotation across the C5'–C7 bond is crucial for generating a pair of atropisomers. Nevertheless, the orientation across the C=C double bond is obviously essential for generating a pair of conformers, for which atropisomerism was not detected in *E*-isomeric pitavastatin and rosuvastatin analogues.

## Supplementary Materials

Supplementary material associated with this article contains ^1^H- and ^13^C-NMR spectra for **P-1**, **P-2**, and **P-3**, table with average values of integrals, which allowed the estimation of mole fractions of the minor conformers and equilibrium constants, and Cartesian coordinates of energy optimized structures for **P-1** at B3LYP/6-311+G(d,p) level. They can be accessed at: http://www.mdpi.com/1420-3049/18/11/13283/s1.
