# Supplementary material for: Conformational Analysis of Geometric Isomers of Pitavastatin Together with Their Lactonized Analogues
Source: Molecules. 2013 Oct 28;18(11):13283–96. doi: 10.3390/molecules181113283 (PMC6270003; doi:10.3390/molecules181113283)
Supplement: Supplementary file 1 [file molecules-18-13283-s001.pdf]

Supplementary Materials

Figure S1. ^{1}H-NMR spectrum of **P-1** in acetone-d_6 at 298 K (600 MHz).

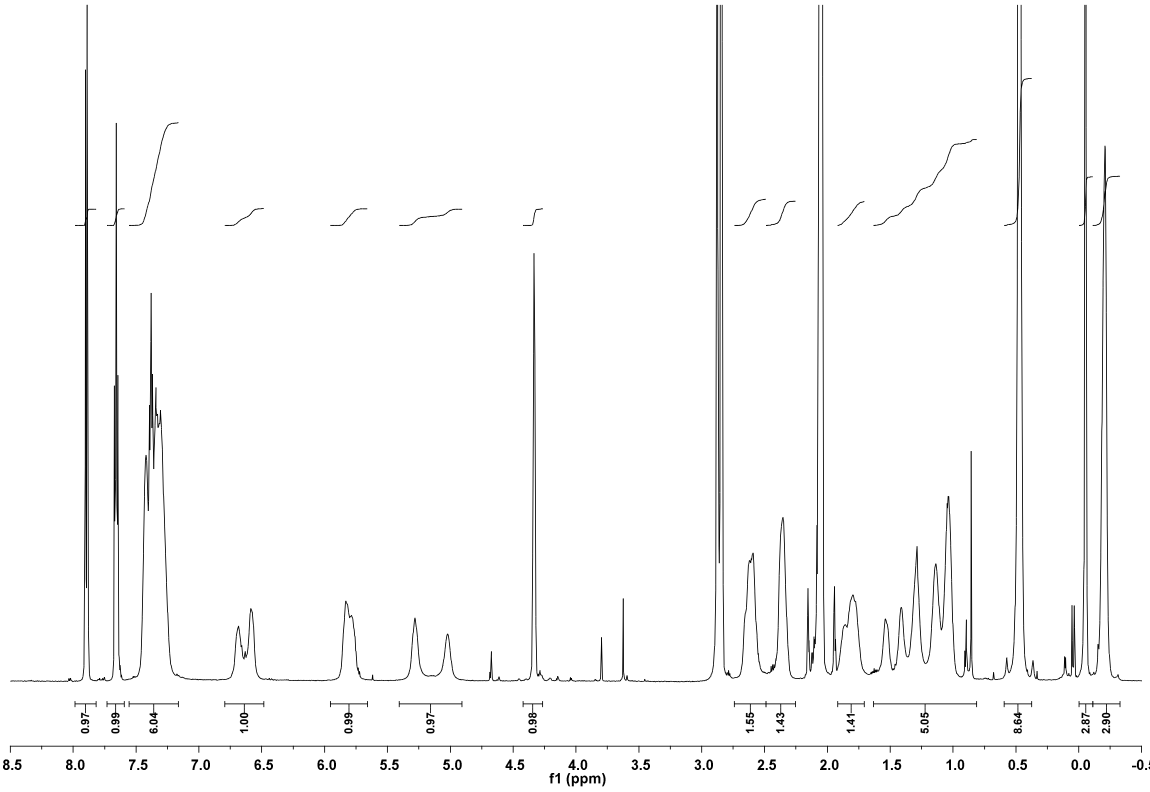

Figure S2. ^{13}C-NMR spectrum of **P-1** in acetone-d_6 at 298 K (75 MHz).

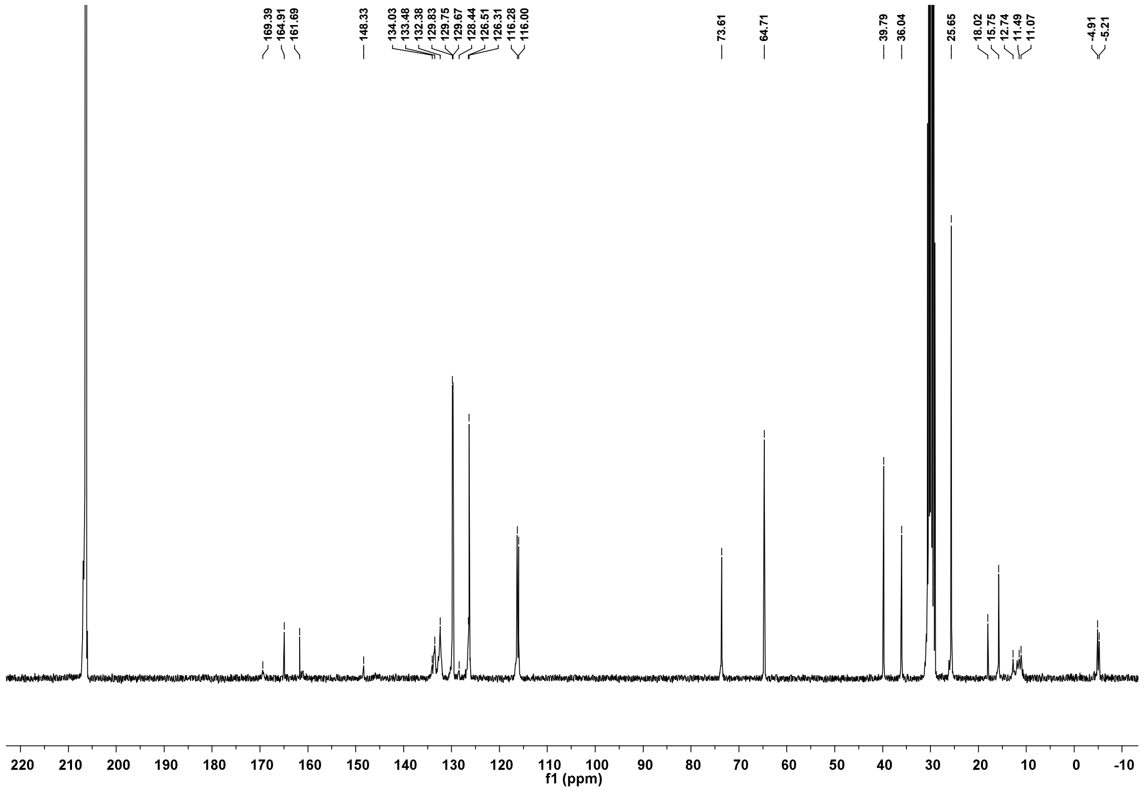

Figure S3. ^{1}H-NMR spectrum of **P-2** in acetone-d_6 at 298 K (600 MHz).

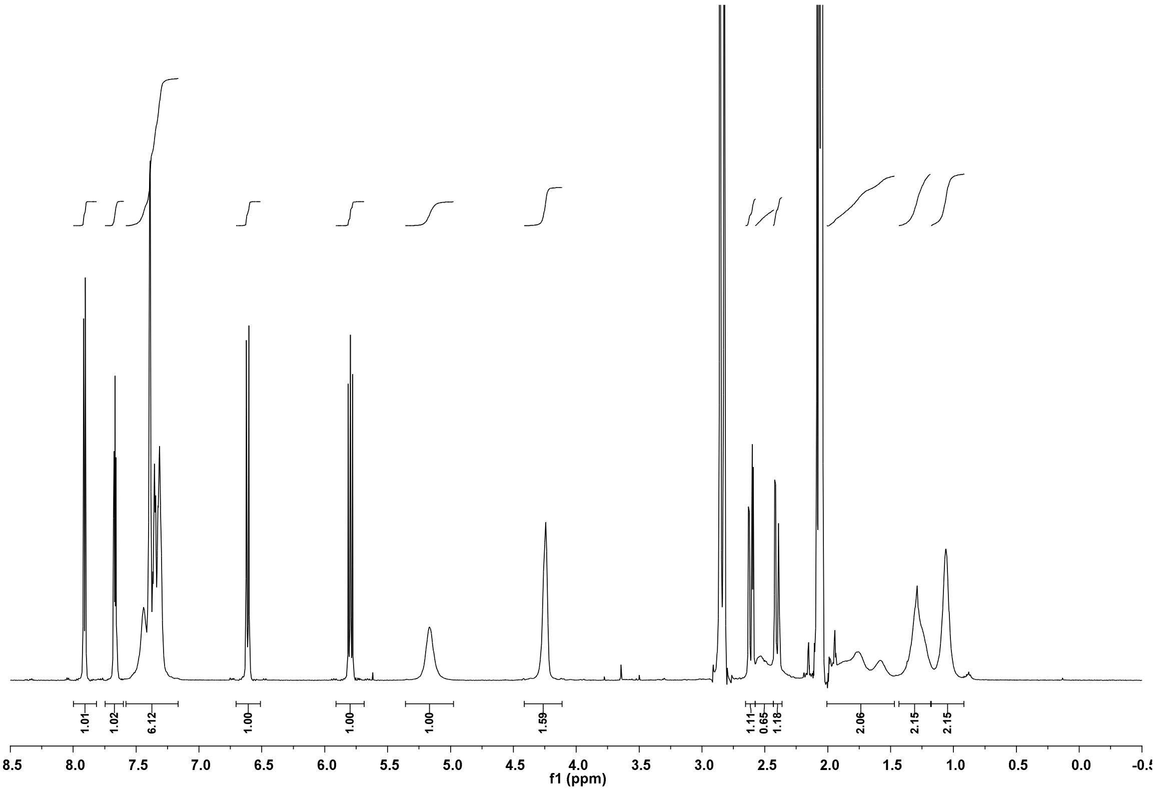

Figure S4. ^{13}C-NMR spectrum of **P-2** in acetone-d_6 at 298 K (75 MHz).

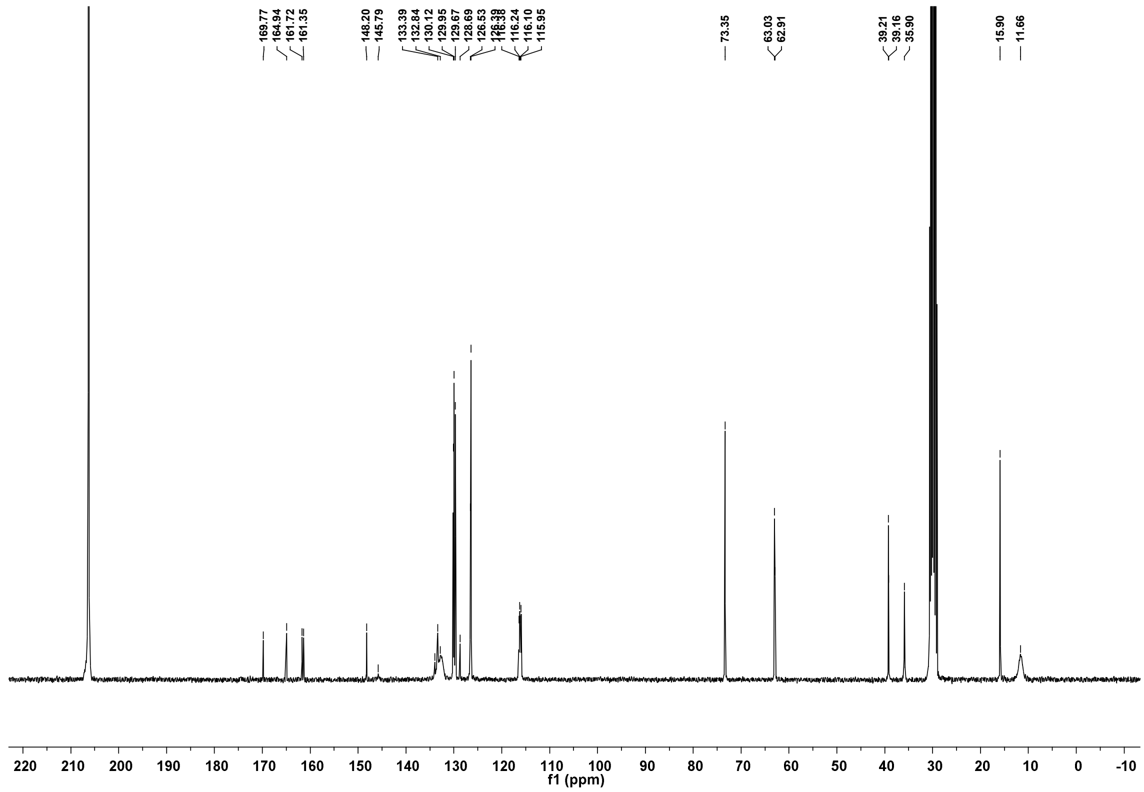

Figure S5. ^{1}H-NMR spectrum of **P-3** in methanol-d_4 at 298 K (600 MHz).

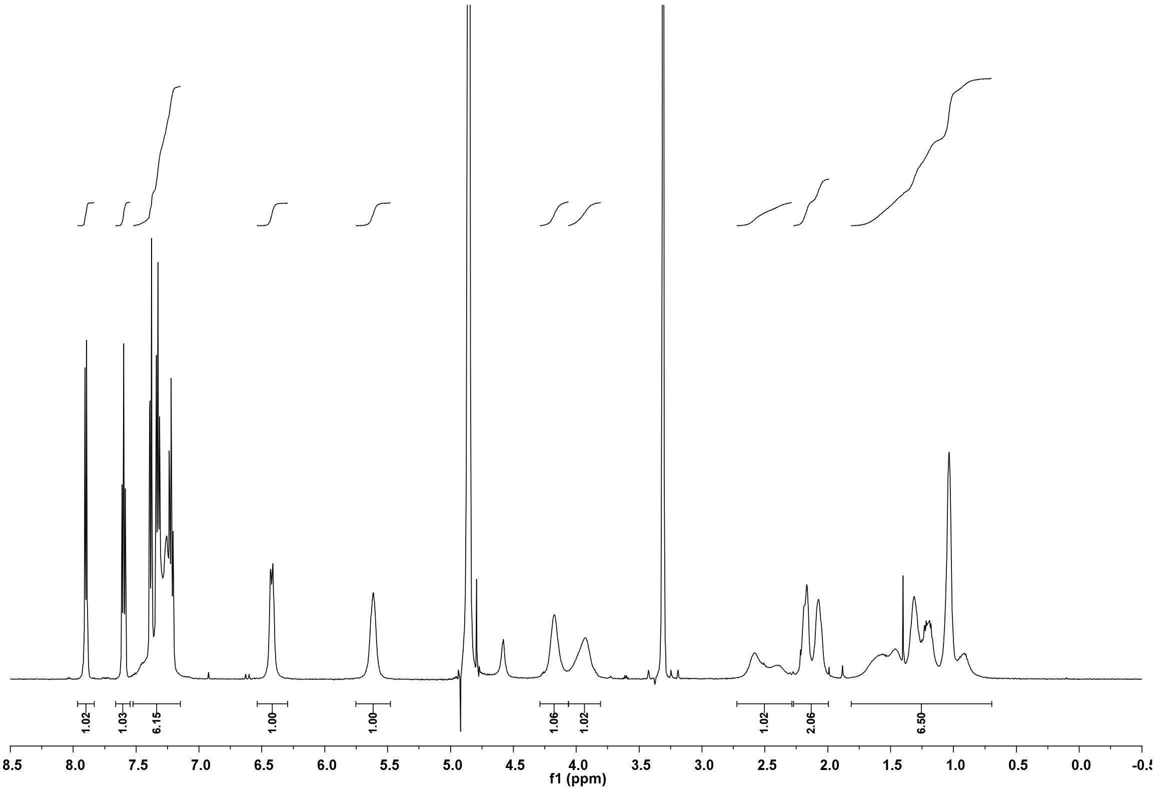

Figure S6. ^{13}C-NMR spectrum of **P-3** in methanol-d_4 at 298 K (75 MHz).

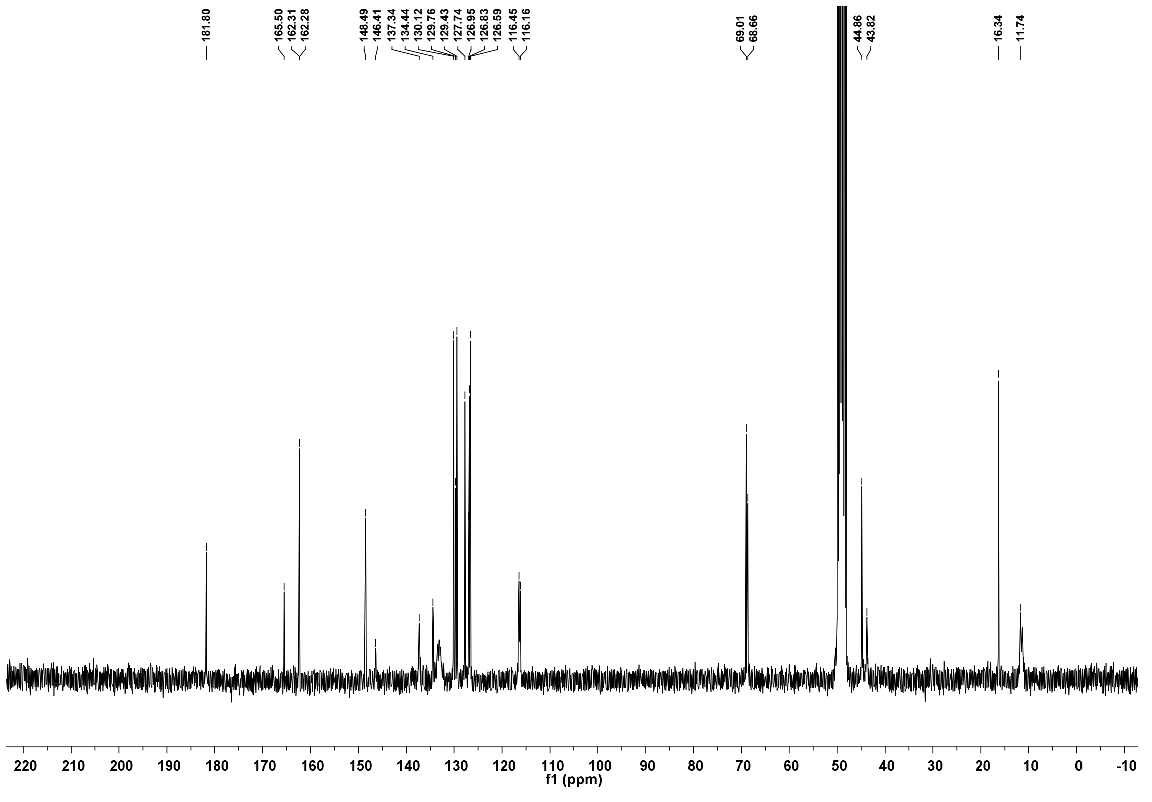

Table S1. Estimation of mole fractions of the minor conformers (m/(M+m)) and equilibrium constants (*K*) based on the average values of integrals of at least two different well-resolved proton signals in ^{1}H NMR spectra for **P-1**, **P-2**, and **P-3**.

		223 K		233 K		243 K		253 K	
	proton	M	m	M	m	M	m	M	m
**P-1**	H7	1.41	1.00	1.37	1.00	1.33	1.00	1.31	1.00
	H6	1.40	1.00	1.37	0.99	1.34	0.99	1.31	1.00
	H5	1.39	0.97	1.35	0.96	1.32	0.97	1.29	0.97
	average	1.40	0.99	1.36	0.98	1.33	0.99	1.30	0.99
	m/(M+m)	**0.414**		**0.419**		**0.426**		**0.432**	
	K=m/M	**0.707**		**0.721**		**0.742**		**0.760**	
**P-2**	H5	1.00	0.53	1.00	0.56	1.00	0.57	1.00	0.59
	H3	1.00	0.53	1.00	0.54	1.01	0.58	1.01	0.59
	average	1.00	0.53	1.00	0.55	1.01	0.58	1.01	0.59
	m/(M+m)	**0.346**		**0.355**		**0.364**		**0.370**	
	K=m/M	**0.530**		**0.550**		**0.572**		**0.587**	
**P-3**	H7	1.00	0.50	1.00	0.52	1.00	0.56	1.00	0.58
	H6	1.02	0.50	1.01	0.52	1.02	0.55	1.03	0.59
	average	1.01	0.50	1.01	0.52	1.01	0.56	1.02	0.59
	m/(M+m)	**0.331**		**0.341**		**0.355**		**0.366**	
	K=m/M	**0.495**		**0.517**		**0.550**		**0.576**	

Cartesian coordinates of energy optimized structures at B3LYP/6-311+G(d.p) level

P-1, energy minimum at $\phi = 122°$

C	-6.03965	1.52876	2.19667
C	-6.50357	0.23718	2.53762
C	-5.90261	-0.87770	2.00745
C	-4.81101	-0.75057	1.11031
C	-4.34375	0.55102	0.76076
C	-4.98441	1.68369	1.32989
N	-4.25680	-1.87975	0.60429
C	-3.22685	-1.80177	-0.21411
C	-2.63800	-0.53900	-0.58964
C	-3.23022	0.62951	-0.12734
C	-1.04548	3.73252	-0.37935
C	-1.53044	2.48606	0.00658
C	-2.70721	1.96687	-0.54486
C	-3.39526	2.72978	-1.49640
C	-2.91928	3.97514	-1.89870
C	-1.74936	4.45490	-1.33061
C	-1.48411	-0.44300	-1.52327
C	-0.25463	-0.94619	-1.37803
C	-2.71374	-3.08703	-0.76002
C	-2.60758	-4.29850	0.15768
C	-3.65463	-4.26905	-0.90704
F	-1.28216	5.66337	-1.71307
C	0.26220	-1.71550	-0.19252
C	1.42014	-1.01890	0.53182
C	2.72358	-1.11705	-0.25139
C	3.04096	-2.59432	-0.47636
C	1.87915	-3.45675	-0.94653
O	0.61323	-3.05965	-0.64695
O	2.04022	-4.50472	-1.50881
O	3.76858	-0.50729	0.49237
Si	4.92780	0.58716	-0.05412
C	5.90578	-0.17383	-1.47534
C	4.07142	2.14713	-0.67962
C	6.01318	0.92069	1.47961
C	6.64108	-0.40098	1.97142
C	5.15110	1.51554	2.61341
C	7.13864	1.91539	1.12237
H	-6.51839	2.40119	2.62660
H	-7.33720	0.13104	3.22277
H	-6.23555	-1.87941	2.25048
H	-4.62772	2.67508	1.08058
H	-0.13925	4.14679	0.04460
H	-0.99329	1.91289	0.75287
H	-4.31061	2.34369	-1.93006
H	-3.44004	4.57124	-2.63767
H	-1.67080	0.14490	-2.41907
H	0.45974	-0.77915	-2.18041
H	-1.93773	-3.00584	-1.50539
H	-1.73619	-4.92806	0.02785
H	-2.94020	-4.14925	1.17763
H	-4.67654	-4.10184	-0.59216
H	-3.52334	-4.89115	-1.78425
H	-0.54682	-1.88613	0.51570
H	1.15464	0.02688	0.70284
H	1.57687	-1.48377	1.51055
H	2.60638	-0.61514	-1.22149
H	3.36627	-3.01234	0.48335
H	3.86087	-2.74198	-1.17966
H	6.39288	-1.10733	-1.18214
H	6.68109	0.51054	-1.83272
H	5.25530	-0.39322	-2.32813
H	3.47761	2.62631	0.10312
H	4.79792	2.87940	-1.04433
H	3.39928	1.92180	-1.51372
H	7.24802	-0.21949	2.86697
H	7.29806	-0.84884	1.21957
H	5.87515	-1.13606	2.23100
H	5.76730	1.68602	3.50483
H	4.71213	2.47814	2.33379
H	4.33756	0.84200	2.89439
H	7.76473	2.10693	2.00217
H	6.74542	2.88089	0.78907
H	7.79542	1.52990	0.33628

P-1, energy minimum at $\phi = 252°$

C	-6.71434	-0.97329	-1.47430
C	-6.59365	-2.37907	-1.56590
C	-5.48904	-3.01386	-1.05462
C	-4.45791	-2.27090	-0.42426
C	-4.58249	-0.85347	-0.31758
C	-5.73304	-0.22736	-0.86676
N	-3.38464	-2.94367	0.06174
C	-2.40166	-2.28013	0.63445
C	-2.40873	-0.84828	0.76323
C	-3.51604	-0.14223	0.31232
C	-4.58979	3.28631	1.53942
C	-4.50589	1.90595	1.37679
C	-3.59625	1.34177	0.47302
C	-2.77175	2.19145	-0.27547
C	-2.85124	3.57329	-0.12791
C	-3.75915	4.09610	0.77961
C	-1.29456	-0.11861	1.43189
C	-0.07307	0.10030	0.94048
C	-1.27487	-3.09642	1.17222
C	-1.54097	-4.51545	1.63780
C	-0.77400	-4.29364	0.37431
F	-3.83902	5.43732	0.93246
C	0.39944	-0.32343	-0.42671
C	1.86170	-0.76846	-0.47534
C	2.82075	0.41358	-0.36948
C	2.50982	1.40092	-1.49473
C	1.03861	1.73643	-1.67464
O	0.12571	0.78174	-1.35552
O	0.65654	2.76834	-2.15695
O	4.15754	-0.04850	-0.48990
Si	5.47319	0.33900	0.49041
C	5.74460	2.20520	0.48101
C	5.12238	-0.21488	2.25933
C	6.93252	-0.61241	-0.28903
C	7.13949	-0.14798	-1.74619
C	6.63365	-2.12669	-0.28374
C	8.22239	-0.34606	0.51654
H	-7.58499	-0.48140	-1.89284
H	-7.37650	-2.95402	-2.04786
H	-5.36547	-4.08833	-1.11542
H	-5.82535	0.84959	-0.81158
H	-5.28350	3.73570	2.23909
H	-5.15108	1.26050	1.96200
H	-2.05532	1.77752	-0.97347
H	-2.21620	4.23400	-0.70483
H	-1.52923	0.30585	2.40626
H	0.62203	0.68350	1.53894
H	-0.52761	-2.54561	1.72519
H	-1.00892	-4.85495	2.51855
H	-2.56274	-4.85817	1.53968
H	-1.29173	-4.48837	-0.55668
H	0.29330	-4.48186	0.36710
H	-0.23761	-1.12181	-0.80294
H	2.04487	-1.48131	0.33260
H	2.06291	-1.28488	-1.41889
H	2.67912	0.91463	0.59798
H	2.84186	0.94636	-2.43553
H	3.05123	2.34027	-1.38298
H	5.94093	2.58240	-0.52601
H	6.59104	2.48658	1.11461
H	4.86700	2.73328	0.86767
H	4.94367	-1.29157	2.31952
H	5.96105	0.02306	2.92046
H	4.24149	0.29150	2.66662
H	7.96830	-0.70169	-2.20419
H	7.38753	0.91604	-1.80530
H	6.24709	-0.32090	-2.35290
H	7.45975	-2.67504	-0.75300
H	6.51957	-2.51903	0.73150
H	5.72128	-2.35788	-0.83927
H	9.06204	-0.89705	0.07586
H	8.13318	-0.67126	1.55772
H	8.49591	0.71363	0.51685
